# Antimicrobial Evaluation of Latex and TLC Fractions from the Leaves of *Aloe adigratana* Reynolds

**DOI:** 10.1155/2020/8312471

**Published:** 2020-03-27

**Authors:** Demoze Asmerom, Gebremedhin Solomon Hailu, Ebrahim M. Yimer, Helen Bitew, Getu Kahsay

**Affiliations:** ^1^Department of Medicinal Chemistry, School of Pharmacy, College of Health Sciences, Mekelle University, P.O. Box 1871, Mekelle, Ethiopia; ^2^Department of Pharmacology, School of Pharmacy, College of Health Sciences, Mekelle University, P.O. Box 1871, Mekelle, Ethiopia; ^3^Department of Pharmacognosy, School of Pharmacy, College of Health Sciences, Mekelle University, P.O. Box 1871, Mekelle, Ethiopia; ^4^Department of Pharmaceutical Analysis & Quality Assurance, School of Pharmacy, College of Health Sciences, Mekelle University, P.O. Box 1871, Mekelle, Ethiopia

## Abstract

**Background:**

The highest prevalence and emergence of microbial infections coupled with the threat of antimicrobial resistance constitute a global concern, which entails searching for novel antimicrobial agents. Medicinal plants are among the major sources of medicines for novel drug discovery. *Aloe adigratana* is one of the endemic *Aloe* species in Ethiopia where the leaf latex of the plant is traditionally used for the treatment of various pathogenic conditions such as wound, dandruff, malaria, and diabetes. In spite of such claims, there was no scientific study done so far. The aim of the current study was, therefore, to evaluate the antimicrobial effect of leaf latex of *A. adigratana* and its thin layer chromatography (TLC) fractions.

**Methods:**

Thin layer chromatography (TLC) separation was employed for isolation of bioactive compounds. Agar well diffusion and microdilution assay method were used to evaluate the antimicrobial actions of the leaf latex and TLC fractions against six bacterial strains and four *Candida* species of reference and clinical isolate microbial strains.

**Results:**

Three major fractions, AA01, AA02, and AA03, were identified by TLC. Among the tested microbial strains, the reference strain of *Staphylococcus aureus* ATCC 29213 (MIC = 0.06 mg/mL) and clinical *Candida krusei* 242/18 (MIC = 0.14 mg/mL) exhibited higher susceptibility towards AA02, while reference strains of *Klebsiella pneumoniae* ATCC 700603 (MIC = 0.19 mg/mL) revealed the highest susceptibility towards AA01. The leaf latex displayed the highest activity against *Staphylococcus aureus* ATCC 29213 and clinical *Candida krusei* 242/18 with a MIC value of 0.19 mg/mL.

**Conclusion:**

The leaf latex and TLC fractions were found to be active against the tested bacterial and *Candida* species. Therefore, this finding supports the traditional claim of *Aloe adigratana* and the need for characterization of the TLC fractions to provide as lead compounds for further comprehensive antibacterial and antifungal activities.

## 1. Introduction

It is clear that antibiotics have become crucial public health medicines since the discovery of penicillin in 1928 [1]. However, the emergence of antimicrobial resistance (AMR) has been increasingly recognized as a serious public health concern worldwide [2]. The flourishing of AMR is mainly associated with overuse and misuse of antibiotics, emerging of resistant species, and the slowdown of antibiotic drug discovery [3]. These days, the pace of the rise of antibiotic use and the discovery of new antibiotic agents is not balanced [4]. Hence, it is undoubtedly necessary to search for new antimicrobial agents to encounter the highest prevalence and emergence of microbial infections coupled with the threat of AMR.

In the majority of developing regions, where modern medicines are unaffordable and scarce, people utilize medicinal plants to meet their primary healthcare needs [5]. In Ethiopia, more than 80% of the population depend on traditional medicines to cure their ailments [6]. The genus *Aloe* is among the commonly utilized remedial plants across the diversified community of the country. In fact, the bitter leaf exudate of different *Aloe* species is broadly used by different communities for various pathological conditions [7]. In Ethiopia there are around 46 species of *Aloe,* of which about 66% are endemic in the country [8]. Several of these endemic species possessed different biological activities, including antimicrobial [9, 10], antitrypanosomiasis [11], antileishmaniasis [12, 13], antimalarial [14–16], wound healing [17], anti-inflammatory [17, 18], anticancer [19], antidiabetic [20], and antioxidant activities [21]. This relevance indicates that *Aloe* is the store house of numerous bioactive compounds [22].

**Figure 1:**
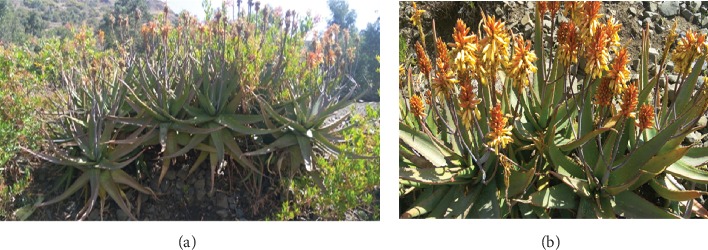
Photograph of *A. adigratana* (taken at Adiaynom, near Adigrat city).

The experimental plant *A. adigratana* (Figure 1), locally known as “*eret*” in Amharic and “*ere habesha*” in Tigrigna, is one of the endemic *Aloe* species in Ethiopia which is specifically found in Adigrat, Tigray region [23]. The local community of Northern Ethiopia planted *A. adigratana* in their farmlands and home gardens to be used for various purposes including management of malaria, wound, dandruff, and diabetes mellitus [18, 24]. Likewise, the people of Southern Ethiopia, Hawassa round district, locally use the fresh leaf exudate of *A. adigratana* for the remedy of malaria and insect bites [25]. *In vivo* and *in vitro* studies also revealed its anti-inflammatory and antioxidant activities, respectively [18, 21]. In spite of such ethnobotanical claims and some scientific studies, neither the antimicrobial effects of the leaf latex nor those of the TLC fractions of *A. adigratana* have been evaluated so far.

Therefore, the aim of the present study was to evaluate the antimicrobial effect of the latex and TLC fractions from the leaf of *A. adigratana* Reynolds against clinical isolates and reference microbial strains.

## 2. Materials and Methods

### 2.1. Chemicals and Equipment

The following chemicals and equipment were used to carry out this study. Methanol and ethyl acetate were purchased from Carlo Erba reagents (Val-de-Reuil, France). Distilled water was purchased from Jouri Labs (Addis Ababa, Ethiopia). Silica gel GF_254_ for TLC was obtained from Merck KGaA (Darmstadt, Germany). The microbial media, Mueller–Hinton agar (MHA), Mueller–Hinton broth (MHB), Sabouraud dextrose agar (SDA), Sabouraud dextrose broth (SDB), and dextrose, were obtained from HiMedia Laboratories (Mumbai, India). The culture medium nutrient agar (NA) was purchased from Thermo Fisher Scientific (Basingstoke, England). Resazurin sodium salt was acquired from Sisco Research Laboratories (Delhi, India). Ceftriaxone disks were acquired from Abtek Biologicals (Liverpool, England), ceftazidime disks from HiMedia Laboratories (Delhi, India), and ketoconazole from Addis Pharmaceutical Factory (Ethiopia). All the solvents used were of analytical grade.

Analytical TLC plate, TLC glass plate, and coating machine were obtained from Merck KGaA (Darmstadt, Germany), vacuum oven from (Daihan Scientific, Seoul, South Korea), and 96-well microtiter plate from Thermo Fisher Scientific (Basingstoke, England).

### 2.2. Collection and Identification of Plant Material

The fresh leaf exudate of *A. adigratana* was collected from “Adiaynom,” situated near Adigrat city, Tigray region, Northern Ethiopia. The plant was identified and authenticated by a botanist from the Department of Biology, College of Computational and Natural Science, University of Gondar, for future reference with voucher number of DA0050/2018.

### 2.3. Preparation of the Latex and Isolation of the TLC Fractions

The leaf latex of *A. adigratana* was collected by cutting the leaves transversally near the base and inclining on a stainless tray. The latex was then dried in shade area for four days at room temperature.

The preparative TLC plate was prepared by using a method described by Kagan and Flythe [26], i.e., suspending silica gel GF_254_ powder and distilled water in a ratio of 1 : 2. The thickened slurry was then poured into the automatic spreading device (adjusted 0.5 mm thickness) and coated in a 20 × 20 cm plate. The plate was air-dried for 30 minutes till it turned white and activated at 100°C for 90 minutes. About 10 *μ*L of latex dissolved in methanol (around 10 mg/mL) was applied using capillary tube, 1.5 cm above the lower edge of the TLC plates, and developed in a solvent tank saturated with ethyl acetate (EtOAc), methanol (MeOH), and water (H_2_O) in a ratio of 77 : 13 : 10 *v/v/v*, respectively [27]. The developed chromatogram was visualized under ultraviolet (UV) light at 254 nm and 366 nm. The bands with major compounds were carefully scrapped off alongside the adsorbent, washed with EtOAc/MeOH (1 : 1), and filtered to yield three major compounds. The purity of the TLC fractions was repeatedly checked using analytical TLC plate. Preparative TLC plates with 0.25 mm thickness were used for further purification of the compounds.

### 2.4. Microorganisms

Six bacterial strains and four *Candida* species were selected based on their public health relevance. The bacterial strains were *S. aureus* ATCC 29213 (reference), *S. aureus* (clinical isolate 184548), *E. coli* ATCC 25922 (reference), *E. coli* (clinical isolate 786824), *K. pneumoniae* ATCC 700603 (reference), and *P. aeruginosa* ATCC 27853 (reference). The fungal strains were clinical *Candida* species including *C. albicans* 240/18, *C. glabrata* 122/18, *C. tropicalis* 12/18, and *C. krusei* 242/18.

### 2.5. Media Preparation and Inoculum Standardization

The fungal and bacterial media were prepared and used according to the manufacturer's instruction and specifications. The bacterial and fungal turbidity of each of the investigated strains were prepared and standardized based on the Clinical and Laboratory Standard Institute (CLSI) guideline [28]. The bacterial strains were subcultured in nutrient agar while the *Candida* species were subcultured in Sabouraud dextrose agar (SDA). The turbidity of the inoculum was similar to the turbidity of 0.5 McFarland standard, which is supposed to contain a bacterial concentration of 1-2 × 10^8^ CFU/mL and fungal concentration of 1–5 × 10^6^ CFU/mL.

### 2.6. Antimicrobial Assay

#### 2.6.1. Agar Well Diffusion

The antimicrobial susceptibility test of the leaf latex and TLC fractions was performed based on previous studies with slight modifications [29, 30]. Once the actively growing bacterial broth culture was standardized to a density of 0.5 McFarland standard, it was streaked on the sterile Mueller–Hinton agar (MHA) plates in 100 mm diameter of sterile Petri dish. The bacteria were then inoculated to the plates using a sterile cotton swab in order to ensure even coverage of the plates and a uniform thick lawn of growth during incubation. The seeded media were allowed to dry at room temperature for 30 minutes.

On each plate, the wells were made with a 6 mm diameter sterilized cork borer and labeled with numbers corresponding to the leaf latex and TLC fractions. The corresponding wells were filled with 50 *μ*L of 200 mg/mL, 100 mg/mL, and 50 mg/mL of the suspensions of the latex dissolved in 2% dimethyl sulfoxide (DMSO). However, 50 mg/mL of TLC fractions dissolved in 2% DMSO, except AA03 which was dissolved in 5% DMSO, was applied to the punched wells. In addition, the standard antibiotic disc of ceftriaxone (30 *μ*g) was used as a positive control for all bacterial strains, except for *P. aeruginosa* ATCC 27853 where ceftazidime (30 *μ*g) was used. Then, the plates were left undisturbed for about 2 hr at room temperature to give enough time to diffuse on the inoculated agar. The plates for the antibacterial study were incubated at 37°C for 24 hours.

Zone of inhibition was measured using zone scale (metal caliper) and reported in millimeter (mm). The experiment was performed in triplicate for each bacterium, and the mean of zones of inhibition was calculated for each sample and the standard antibiotics.

To determine the antifungal activities of the samples, both the leaf latex and TLC fractions were dissolved in DMSO, and the zone of inhibition was determined by agar well diffusion method similar to the procedure stated above. The only difference was that the culture medium for *Candida* was MHA supplemented with 2% glucose and 0.5 *μ*g/mL methylene blue [31]. The positive control used was 50 *μ*g ketoconazole dissolved in DMSO.

#### 2.6.2. Broth Microdilution Assay

The latex and TLC fractions that displayed antimicrobial activity by agar well diffusion method were subjected to serial microdilution technique to determine the minimum inhibitory concentration (MIC) using the growth indicator, resazurin sodium salt (Alamar blue) [32, 33].

Under aseptic conditions, the 96-well microtiter plates were labeled (columns 1–12 and rows A–H), and a volume of 100 *μ*L Mueller–Hinton broth (MHB) was dispensed to all wells of microtiter plates. Next, a volume of 100 *μ*L of stock suspension (100 mg/mL) of leaf latex and TLC fractions (25 mg/mL) was pipetted into the first column of the microtiter plate. Twofold serial dilution of either leaf latex or TLC fractions (until the 10^th^ column) was performed using a multichannel micropipette. Then, 100 *μ*L of mixed solution of the test material and the MHB was discarded from the 10^th^ column so that each well contained 100 *μ*L of test material in serially descending concentrations ranging from 100 to 0.19 mg/mL for the leaf latex and from 25 to 0.04 mg/mL for TLC fractions. Afterwards, a 0.5 McFarland turbidity standard bacterial suspension was adjusted and further diluted to 1 : 100 (5 × 10^6^ CFU/mL) with saline (0.85%). Within 15 minutes of the preparation of the inoculum, 20 *μ*L of bacterial suspension was added to each well, except column 12, to achieve a final concentration of 1 × 10^5^ CFU/mL. Each microtiter plate had a set of two controls: the 11^th^column with all solution constituents except the leaf latex and TLC fractions was used as color contrast and growth control, while the 12^th^ column with all solution constituents except bacterial suspension was used as sterility control; instead, 10 *μ*L of nutrient broth was added. Then, each plate was wrapped loosely with parafilm to avoid dehydration of the bacteria, and the plates were incubated at 35–37°C for 24 hrs [34].

Finally, 30 *μ*L of resazurin solution (0.015% (w/v)) was added to each well and further incubated for 2–4 hr for observation of color change visually. After incubation, any color change observed from blue/purple to pink or colorlessness was taken as positive (no inhibition). The lowest concentration of the plant leaf latex or TLC fractions at which no color change occurred was recorded as the MIC value. All the experiments were performed in duplicate, and the average value was taken for MIC of leaf latex and each TLC fraction [32].

A similar procedure was adopted for determination of the MIC of the latex and TLC fractions against *Candida* species, except that Sabouraud dextrose broth was used for growth of *Candida* species instead of Mueller–Hinton broth.

#### 2.6.3. Determination of Minimum Bactericidal Concentration (MBC) and Fungicidal Concentration (MFC)

The MBC and MFC were determined by subculturing of the contents of the wells from MIC and above concentrations of the serial broth microdilution. The sample containing well that showed no visible growth was subcultured, spreading a loopful of suspension evenly over a divided plate on MHA medium for bacteria or MHA supplemented with 2% glucose and 0.5 *μ*g/mL methylene blue for *Candida* species, and was incubated at 37°C for 24 hrs. The highest dilution that exhibited no growth was considered as MBC for bacterial strains and MFC for fungal strains [34].

### 2.7. Statistical Analysis

Statistical Package for Sciences (SPSS) version 21 was used for statistical analysis. The data were expressed as mean ± standard error of the mean (SEM). Differences between means of all parameters were carried out by using one-way analysis of variance (ANOVA). Subsequently, the post hoc Tukey test was used to determine the level of significance, and *P* < 0.05 was considered statistically significant.

## 3. Results and Discussion

The threat of AMR, the emergence of new resistant microbial strains and adverse drug reactions, and the need for prolonged therapy in some infectious conditions dictate further evaluation of medicinal plants and their derivatives for antimicrobial effect. Hence, this study was carried out to investigate the activity of leaf latex and TLC fractions of *A. adigratana* against different bacterial and fungal strains.

In the present study, the leaf latex and the TLC fractions of *A. adigratana* showed a broad-spectrum antimicrobial activity. This finding is in line with previous works that indicated that most *Aloe* species possessed antimicrobial activities [22]. In both Gram-positive and Gram-negative bacterial strains, the clinical isolate strains were more resistant than the reference strains to the positive control (ceftriaxone) and all tested samples. This might be due to their past exposure to antibiotics, because microorganisms that are originally susceptible to antibiotic agents can develop resistance with prolonged antibiotic experience [35], thereby reducing the access of the leaf latex and the TLC fractions to the target sites via different mechanisms of resistance [36].

### 3.1. Antibacterial Activity of the Leaf Latex of *A. adigratana* by Agar Well Diffusion

The antibacterial effect of the leaf latex of *A. adigratana* by agar well diffusion increased with dose as shown in Table 1. The average mean zone of inhibition was significantly different (*P* < 0.01) for leaf latex at 50 mg/mL, except *S. aureus* (isolate 184548), *E. coli* ATCC 25922 (reference), and *K. pneumoniae* ATCC 700603 (reference), compared to that at 100 mg/mL against the growth of each test bacterium. Similarly, 100 mg/mL leaf latex concentration had significantly different (*P* < 0.01) zone of inhibition compared to 200 mg/mL for all bacterial species except *E. coli* (clinical isolate 786824). Comparison of the different concentrations of leaf latex with standard positive control disk showed significantly different zone of inhibition (*P* < 0.01), except for clinically isolated *S. aureus* (isolate 184548) at 100 mg/mL. Moreover, in clinically isolated *E. coli* (isolate 786824) the average inhibition of the different concentrations of the leaf latex was significantly different (*P* < 0.01).

Among the test bacteria, the maximum average zone of inhibition at 200 mg/mL concentration was 17.76 mm (*P. aeruginosa* ATCC 27853) followed by 16.5 mm for *S. aureus* ATCC 29213 (reference). Of all the bacterial strains, *E. coli* (clinical isolate 786824) and *K. pneumoniae* ATCC 700603 (reference) were less susceptible with an average zone of inhibition of 13.03 and 13.9 mm at 200 mg/mL concentration of leaf latex, respectively.

The ability of the leaf latex to inhibit the clinical isolate pathogens was very important, because the clinical isolate *E. coli* was found resistant to the positive control ceftriaxone, which is the mainstay of therapy for infections attributed to *E. coli* [37]. *Enterobacteriaceae* species including *E. coli* are among the top prioritized species resistant to the third generation cephalosporins and have been given priority to be investigated by the research community to tackle this global resistance [38].

Prior studies concerning the *in vitro* antibacterial activity of the leaf extracts of *A*. *vera* possessed antibacterial activities against both Gram-positive and Gram-negative bacteria including *S. aureus*, *Bacillus subtilis*, *Salmonella* typhi, *P. aeruginosa*, *E. coli,* and *K. pneumoniae* [39]. Moreover, the leaf latex of Ethiopian *Aloe* species showed promising antibacterial activity against *S. aureus*, *E. coli*, *S.* typhi, *Shigella* spp., *V. cholerae*, and *Salmonella* spp. [9, 10]. This could be due to the presence of bioactive compounds in *Aloe* species including anthraquinones, such as aloin A/B, homonataloin, aloe emodin, and chrysophanol, which had been reported to exhibit antibacterial activity [40]. Thus, the presence of anthraquinones and other constituents might be responsible for the antibacterial activities of leaf latex of *A. adigratana* [18].

### 3.2. Antifungal Activity of the Leaf Latex of *A. adigratana* by Agar Well Diffusion Method

Fungal infections are also increasing from time to time, particularly following the advent of human immune deficiency virus (HIV) infection and the widespread of azole-resistant strains which have contributed to significant illnesses and death [41]. Considering these problems, the antifungal activity of leaf latex of *A. adigratana* was also undertaken. Interestingly, the leaf latex inhibited all tested *Candida* species in dose-dependent fashion presented in Table 2. This might be due to plants are endowed with various active constituents that inhibit fungal growth [42]. The mean zone of inhibition of all concentrations of leaf latex showed statistically significant difference (*P* < 0.01) when compared with ketoconazole, except for *C. krusei* 242/18 which showed comparable activity at 400 mg/mL and 200 mg/mL. The average zone of inhibition at 200 mg/mL for each concentration was significantly different (*P* < 0.01) compared to that at 400 mg/mL against all *Candida* species. Similarly, the mean zone of inhibition at 100 mg/mL of each concentration showed a significant difference (*P* < 0.01) when compared with that at 200 as depicted in Table 2.

### 3.3. Isolation of TLC Fractions

**Figure 2:**
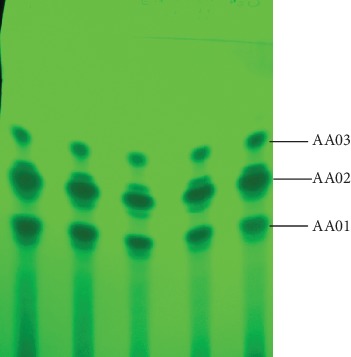
UV visualization of TLC fractions under UV light of 254 nm.

Based on the encouraging antimicrobial activity of the leaf latex of *A. adigratana* by agar well diffusion and broth microdilution methods, it was further analyzed on preparative TLC for phytochemical investigation with the aim of finding more effective antibacterial and antifungal phytocompounds. Treatment of the leaf latex of *A. adigratana* under a solvent system of EtOAc/MeOH/H_2_O (77 : 13 : 10) yielded three major compounds assigned as AA01, AA02, and AA03 (Figure 2). TLC fraction 1 (AA01) was obtained as a yellow solid with R_*f*_ value of 0.33, while TLC fractions 2 (AA02) and 3 (AA03) were also obtained as yellow solids with R_*f*_ values of 0.48 and 0.68, respectively, in the same solvent system.

### 3.4. Antibacterial and Antifungal Activities of the TLC Fractions by Agar Well Diffusion

Before proceeding to the microdilution assay, the TLC fractions were initially assessed for their antibacterial and antifungal activity at a concentration of 50 mg/mL by agar well diffusion method as shown in Tables 3 and 4. The mean comparison of the TLC fractions at similar concentration differs significantly (*P* < 0.01), except for AA01 and AA02 which possessed comparable activity against all tested bacterial strains. However, the mean comparison with positive control was significant (*P* < 0.01) with all the tested bacterial strains. Like the leaf latex, all the TLC fractions revealed a wide spectrum of activity against all tested bacterial strains. AA01 and AA02 exhibited the highest zone of inhibition against the reference *S. aureus* ATCC 29213. On the other hand, the clinical *S. aureus* was more susceptible to AA01 and AA02 than the positive control, ceftriaxone.

The TLC fractions also hindered the growth of all clinical isolate *Candida* pathogens, which are highly associated with immunocompromised patients [43]. The mean zone of inhibition of AA01 compared to AA02 and AA03 was statistically significant (*P* < 0.01); however, AA01 and AA02 against *C. tropicalis* and *C. krusei* have a comparable zone of inhibition. Furthermore, the observed zone of inhibition of the TLC fractions at the tested concentration was statistically different compared to that of positive control (*P* < 0.01) against all *Candida* species. However, at a similar concentration, TLC fraction 3 (AA03) was less active against all tested bacterial and fungal species than AA01, AA02, and positive control. Therefore, the slightly lower zone of inhibition of AA03 against the tested microbial strains might be associated with the less diffusible nature of the compound in aqueous surfaces of the agar plate [44].

### 3.5. Minimum Inhibitory/Bactericidal Concentration (MIC/MBC) of Leaf Latex and TLC Fractions of *A. adigratana*

The leaf latex and TLC fractions that showed activity by qualitative assay (agar well diffusion) were further subjected to quantitative assay (microdilution technique) in order to obtain more information on the potency of the leaf latex and the TLC fractions. The leaf latex and AA02 were found most effective against Gram-positive *S. aureus* ATCC 29213 (Table 5). Particularly, AA02 was the most potent compound against the reference *S. aureus* ATCC 29213 with MIC value of 0.06 mg/mL. This sensitivity of *S. aureus* could be due to the lack of the outer membrane in Gram-positive which acts as the main permeability barrier in the case of Gram-negative bacteria [45]. As described elsewhere [46], not only is *S. aureus* the causative agent of the lethal hospital-related infections, but it also results in hundreds of thousands of deaths per year largely due to an increase in multidrug resistance (MDR) to the bacteria worldwide [47].

Although deadly Gram-negative bacterial species such as *K. pneumoniae* ATCC 700603 and *P. aeruginosa* ATCC 27853 have developed MDR against most of the currently existing antibiotics [48], their growth was suppressed by TLC fractions of AA01 and AA02. The activity of the TLC fractions against clinical *E. coli* ATCC 25922 was comparatively lower than that of the leaf latex. This might be due to the synergy of the leaf latex constituents against the clinical *E. coli* (clinical isolate 786824). In the current study, compounds obtained from *Aloe* species showed attractive antimicrobial activity. For example, aloin A/B isolated from *A. sinana*, *A. trichosantha*, and *A. trigonantha* showed antibacterial activity against *E. coli*, *S.* typhi Type 2, *Shigella*, *S. aureus*, and *V. cholerae* [9, 10]. This suggests that the chemical structure of the TLC fractions of *A. adigratana* might be under a similar class of bioactive heterocyclic scaffolds of these *Aloe* species.

### 3.6. Minimum Inhibitory/Fungicidal Concentration of the Leaf Latex of *A. adigratana* and TLC Fractions

The antifungal activity of the leaf latex and TLC fractions against the clinical isolate *Candida* species was valuable because these strains are reported to be remarkably resistant to the commonly used antifungal agents [49]. Previous studies regarding the antifungal activity of *Aloe* species against clinical isolate fungal species are sporadic. However, there are some literature works consistent with the findings of the current study on reference *C. albicans* and other fungal species. For example, extracts from *A. barbadensis* Mill, *A. excelsa*, and *A. secundiflora* inhibited the growth of *C. albicans*, *C. glabrata*, *C. tropicalis*, *Aspergillus flavus*, *Aspergillus glaucus*, *Trichophyton mentagrophytes*, and *Trichophyton rubrum* [50–52]. More interestingly, indigenous *Aloe* species like *A. elegans*, *A. trichosantha*, and *A. trigonantha* also possessed antifungal activities against reference strains of *C. albicans*, *Aspergillus niger*, *Penicillium funiculosum*, and *Penicillium notatum* [9, 53, 54].

Concerning the antifungal activity of the TLC fractions, *C. krusei* 242/18 showed the highest sensitivity to AA02 with MIC 0.14 mg/mL and MFC 1.56 mg/mL as illustrated in Table 6. This result was promising for further antifungal investigation because *C. krusei* is intrinsically resistant to the widely prescribed antifungal agent, fluconazole [31]. Furthermore, the strain of *C. albicans* 240/18, the most prominent pathogen, showed similar susceptibility to AA02 with MIC 0.39 mg/mL and MFC 1.56 mg/mL. Even though the antifungal activity of the compounds isolated from *Aloe* species against clinical isolate *Candida* species is rare, the available reports indicated that aloin and 7-O-methylaloeresin isolated from the leaf latex of *A*. *harlana* and *A*. *sinana* inhibit the growth of standard *C. albicans*, *P*. *funiculosum*, and *P. notatum* [9, 10].

The TLC fraction AA03 (relatively nonpolar) demonstrated slightly lower activity against bacterial strains and conversely showed better effect against *Candida* species. The highest activity was revealed on *C. glabrata* 122/18, among the major causative agents of oropharyngeal, esophageal, vulvovaginal, and urinary tract candidiasis and other systemic candida infections [55], with MIC of 0.19 mg/mL and MFC of 3.14 mg/mL, followed by *C. krusei* 242/18 with MIC of 0.2 mg/mL and MFC of 6.25 mg/mL.

### 3.7. Bactericidal/Bacteriostatic and Fungicidal/Fungistatic Nature of the Leaf Latex of *A. adigratana* and Its TLC Fractions

The bactericidal and bacteriostatic nature of the latex and TLC fractions was also evaluated because there are certain circumstances where cidal agents are preferable over static agents and vice versa. For instance, immunosuppressed individuals and patients who have endocarditis and meningitis favor cidal agents to definitely clear the organism from the infected site [56, 57]. According to Kone and his colleagues [58], a given antibacterial substance is said to be bactericidal if the ratio of MBC/MIC is  ≤ 4 and bacteriostatic if MBC/MIC > 4. Hence, the leaf latex was bacteriostatic against almost all bacterial strains except reference *S. aureus*. Nonetheless, the TLC fractions presented varying effect against the bacterial strains, which means the cidal or static nature of the tested samples is not uniform. This could be due to the fact that a given antibacterial agent might be bactericidal to one organism but bacteriostatic to another microorganism. For instance, chloramphenicol is generally considered as bacteriostatic, but bactericidal against *Streptococcus pneumoniae* and *Hemophilus influenzae* at clinically achievable concentration [56]. Likewise, the leaf latex and the TLC fractions of *A. adigratana* revealed both bacteriostatic and bactericidal nature for the tested bacterial strains.

In *Candida* species, all tested samples except *C. krusei* 242/18 possessed fungicidal activity because an agent was regarded as fungicidal if the MFC to MIC ratio was ≤4 and fungistatic if the ratio was >4 [59]. This suggested that the leaf latex and its TLC fractions could be a clue for further antifungal study to be used by both immunocompetent and immunosuppressed individuals.

## 4. Conclusion and Recommendation

TLC fractionation of leaf latex of *A. adigratana* Reynolds yielded three major fractions, AA01, AA02, and AA03. The leaf latex, AA01, AA02, and AA03, displayed relevant activity against clinical isolates and reference strains. The antimicrobial constituents of leaf latex *A. adigratana* are in part or in whole AA01, AA02, and AA03. In particular, AA02 showed the highest activity against the reference *S*. *aureus* and clinical *C*. *krusei* 242/18. The inhibitory effects of TLC fractions against those microorganisms have indicated the activity of *A. adigratana* Reynolds against various infections caused by those strains. Therefore, this finding supports the traditional claim that the plant can be utilized for antimicrobial applications. Characterization of the TLC fractions is required to provide lead compounds which will be used for further comprehensive antibacterial and antifungal activities.

## Figures and Tables

**Table 1:** Zone of inhibition of leaf latex of *A. adigratana* at different concentrations against bacterial strains.

Bacterial strains	Diameter of zone of inhibition (mm)				
	Latex (mg/mL)			Ceftriaxone 30 *µ*g	DMSO
	200	100	50		
*S. aureus* ATCC 29213 (reference)	16.5 ± 0.07^a2b2c2A1B2C2D1E2^	13.2 ± 0.23^a2c2C2E2^	11.2 ± 0.18^a2B2C2D1^	23.4 ± 0.18^A2B2C2^	0
*S. aureus* (isolate 184548)	15.4 ± 0.08^a2b2c1C2D2E2^	12.2 ± 0.29^B2C2D1E2^	11.14 ± 0.44^B1C2D1^	12.3 ± 0.27^B2C2E2^	0
*E. coli* ATCC 25922 (reference)	15.2 ± 0.22^a1b2c2C2D2E2^	12.5 ± 0.12^a2c2C2D1E2^	9.02 ± 0.47^a2D2^	24.6 ± 0.35^C2E2^	0
*E. coli* (isolate 786824)	13.03 ± 0.28^a2b2c2D2^	9.88 ± 0.11^a2D2^	7.9 ± 0.36^a2D2E2^	0.00 ± 0.00^E2^	0
*P. aeruginosa* ATCC 27853 (reference)	17.7 ± 0.23^a2b2c2E2^	14 ± 0.3^a2c1E2^	12.8 ± 0.29^a2E2^	29.7 ± 0.12^*∗*^	0
*K. pneumoniae* ATCC 700603 (reference)	13.9 ± 0.2^a2b2c2^	10.8 ± 0.22^a2^	10.2 ± 0.56^a2^	22.8 ± 0.2	0

Values are expressed as mean ± SEM (*n* = 3); analysis was carried out with one-way ANOVA followed by post hoc Tukey test. Across rows: ^a^compared to positive control, ^b^to 100 mg/mL, and ^c^to 50 mg/mL. Across columns: ^A^compared to *S. aureus* (isolate 184548), ^B^to *E. coli* ATCC 25922, ^C^to *E. coli* (isolate 786824), ^D^to *P. aeruginosa* ATCC 27853, and ^E^to *K. pneumoniae* ATCC 700603. ^1^*P* < 0.05, ^2^*P* < 0.01. 0 means that the negative control has shown no antibacterial activity. ^*∗*^Ceftazidime 30 *µ*g.

**Table 2:** Zone of inhibition of leaf latex of *A. adigratana* at different concentrations against *Candida* species.

*Candida* species	Diameter of zone of inhibition (mm)				
	Latex (mg/mL)			Ketoconazole 50 *µ*g	DMSO
	400	200	100		
*C. albicans* 240/18	17.85 ± 0.4^a2b2c2C2^	14.48 ± 0.26^a2b2C2^	12.25 ± 0.2^a2C2^	24.02 ± 0.11^A2C2^	0
*C. glabrata* 122/18	17.46 ± 0.12^a2b2c2C2^	14.9 ± 0.11^a2b2C2^	12.3 ± 0.16^a2C2^	21.2 ± 0.09^B2^	0
*C. tropicalis* 12/18	18.2 ± 0.18^a2b2c2C2^	14.6 ± 0.32^a2b2C2^	12 ± 0.08^a2C2^	23.1 ± 0.48^C2^	0
*C. krusei* 242/18	22.78 ± 0.44^b2c2^	19.3 ± 0.23^b2^	16.4 ± 0.23^a1^	21.28 ± 0.24	0

Values are expressed as mean ± SEM (*n* = 3); analysis was carried out with one-way ANOVA followed by post hoc Tukey test. Across rows: ^a^compared to positive control, ^b^to 200 mg/mL, and ^c^to 100 mg/mL. Across columns: ^A^compared to *C. glabrata*, ^B^to *C. tropicalis*, and ^C^to *C. krusei*. ^1^*P* < 0.05, ^2^*P* < 0.01. 0 means that the negative control has shown no antifungal activity.

**Table 3:** Zone of inhibition of TLC fractions of *A. adigratana* at 50 mg/mL against bacterial strains.

Bacterial strains	Diameter of zone of inhibition (mm)				
	AA01	AA02	AA03	Ceftriaxone 30 *μ*g	DMSO
*S. aureus* ATCC 29213 (reference)	17.36 ± 0.18^a2c2A2B2C2D2E2^	17.76 ± 0.14^a2c2A2B2C2D2E2^	11.27 ± 0.14^a2E2^	23.03 ± 0.23^A2B2C2^	0
*S. aureus* (isolate 184548)	14.7 ± 0.48^a2c2^	15.1 ± 0.16^a2c2B2C2D2^	10.7 ± 0.25^a2E2^	12.28 ± 0.22^B2C2E2^	0
*E. coli* ATCC 25922 (reference)	14.01 ± 0.28^a2c2^	13.8 ± 0.23^a2c2D1E1^	11.161 ± 0.16^a2E2^	25.58 ± 0.36^C2E2^	0
*E. coli* (isolate 786824)	13.47 ± 0.15^a2c2^	14.15 ± 0.15^a2c2E2^	10.77 ± 0.37^a2E2^	0.00 ± 0.00^E2^	0
*P. aeruginosa* ATCC 27853 (reference)	13.35 ± 0.17^a2c2^	13.23 ± 0.13^a2^	10.2 ± 0.4^a2^	28.8 ± 0.22^*∗*^	0
*K. pneumoniae* ATCC 700603 (reference)	14.06 ± 0.31^a2c2^	14.99 ± 0.07^a2c2E2^	8.89 ± 0.48^a2^	23.33 ± 0.16	0

Values are expressed as mean ± SEM (*n* = 3); analysis was carried out with one-way ANOVA followed by post hoc Tukey test. Across rows: ^a^compared to positive control, ^b^to AA02, and ^c^to AA03. Across columns: ^A^compared to *S. aureus* (isolate 184548), ^B^to *E. coli* ATCC 25922, ^C^to *E. coli* (isolate 786824), ^D^to *P. aeruginosa* ATCC 27853, and ^E^to *K. pneumoniae* ATCC 700603. ^1^*P* < 0.05, ^2^*P* < 0.01. 0 means that the negative control has shown no antibacterial activity. ^*∗*^Ceftazidime 30 *µ*g.

**Table 4:** Zone of inhibition of the TLC fractions of *A. adigratana* at 50 mg/mL against fungal strains.

*Candida* species	Diameter of zone of inhibition (mm)				
	AA01	AA02	AA03	Ketoconazole 50 *µ*g	DMSO
*C. albicans* 240/18	14.01 ± 0.11^a2b2c2^	15.3 ± 0.15^a2c2A2B2^	9.91 ± 0.22^a2C1^	24.9 ± 0.22^A2C2^	0
*C. glabrata* 122/18	14.55 ± 0.16^a2b2c2B1^	13.4 ± 0.17^a2c2B2C2^	10.14 ± 0.29^a2^	22.5 ± 0.21^B2^	0
*C. tropicalis* 12/18	13.8 ± 0.11^a2c2^	14.37 ± 0.19^a2c2C1^	11.01 ± 0.39^a2^	24.27 ± 0.035^C2^	0
*C. krusei* 242/18	14.38 ± 0.19^a2c2^	15.1 ± 0.12^a2c2^	11.28 ± 0.14^a2^	22.26 ± 0.4	0

Values are expressed as mean ± SEM (*n* = 3); analysis was carried out with one-way ANOVA followed by post hoc Tukey test. Across rows: ^a^compared to positive control, ^b^to AA02, and ^c^to AA03. Across columns: ^A^compared to *C. glabrata*, ^B^to *C. tropicalis*, and ^C^to *C. krusei*. ^1^*P* < 0.05,^2^*P* < 0.01. 0 means that the negative control has shown no antifungal activity.

**Table 5:** MIC and MBC (mg/mL) of leaf latex and TLC fractions of *A. adigratana* against bacterial strains.

Bacterial strains	Assay used	Latex	AA01	AA02	AA03
*S. aureus* ATCC 29213 (reference)	MIC	0.19 ± 0.00	0.29 ± 0.1	0.06 ± 0.025	3.13 ± 0.25
	MBC	0.19 ± 0.00	3.125 ± 0.00	0.78 ± 0.00	12.5 ± 0.00
*S. aureus* (isolate 184548)	MIC	0.58 ± 0.19	0.14 ± 0.05	0.14 ± 0.05	6.25 ± 0.02
	MBC	3.125 ± 0.00	0.29 ± 0.1	0.29 ± 0.00	25 ± 0.00
*E. coli* ATCC 25922 (reference)	MIC	0.29 ± 0.1	0.39 ± 0.00	0.39 ± 0.00	0.39 ± 0.00
	MBC	12.5 ± 0.00	6.25 ± 0.00	6.25 ± 0.00	6.25 ± 0.00
*E. coli* (isolate 786824)	MIC	1.56 ± 0.00	2.34 ± 0.75	3.125 ± 0.78	1.56 ± 0.01
	MBC	6.25 ± 0.00	12.5 ± 0.00	12.5 ± 0.00	25 ± 0.00
*P. aeruginosa* ATCC 27853 (reference)	MIC	1.56 ± 0.01	0.78 ± 0.02	1.17 ± 0.35	3.12 ± 0.02
	MBC	25 ± 0.00	12.5 ± 0.00	12.5 ± 0.00	12.5 ± 0.00
*K. pneumoniae* ATCC 700603 (reference)	MIC	0.78 ± 0.00	0.19 ± 0.02	0.78 ± 0.00	0.78 ± 0.00
	MBC	25 ± 0.00	12.5 ± 0.00	12.5 ± 0.00	12.5 ± 0.00

MIC: minimum inhibitory concentration, MBC: minimum bactericidal concentration. The values are the average of duplicate tests (mean ± SEM).

**Table 6:** MIC and MFC (mg/mL) of leaf latex and TLC fractions of *A. adigratana* against bacterial strains.

*Candida* species	Assay used	Latex	AA01	AA02	AA03
*C. albicans* 240/18	MIC	0.78 ± 0.00	3.14 ± 0.00	0.39 ± 0.00	1.56 ± 0.001
	MFC	3.14 ± 0.00	3.27 ± 0.00	1.56 ± 0.00	1.56 ± 0.00
*C. glabrata* 122/18	MIC	3.13 ± 0.02	0.2 ± 0.07	0.39 ± 0.00	1.56 ± 0.02
	MFC	3.14 ± 0.00	0.78 ± 0.00	1.56 ± 0.00	3.14 ± 0.00
*C. tropicalis* 12/18	MIC	3.13 ± 0.02	0.39 ± 0.00	0.78 ± 0.00	1.56 ± 0.01
	MFC	6.25 ± 0.00	0.39 ± 0.00	1.56 ± 0.01	3.14 ± 0.00
*C. krusei* 242/18	MIC	0.19 ± 0.00	0.2 ± 0.00	0.142 ± 0.05	0.2 ± 0.02
	MFC	1.56 ± 0.00	3.14 ± 0.00	1.56 ± 0.00	1.56 ± 0.00

MIC: minimum inhibitory concentration, MFC: minimum fungicidal concentration. The values are the average of duplicate test (mean ± SEM).

## Data Availability

The data used to support the findings of this study are available from the corresponding author upon request.

## Abbreviations

AMR: Antimicrobial resistance
ATCC: American Type Culture Collection
CFU: Colony forming unit
DMSO: Dimethyl sulfoxide
EtOAc: Ethyl acetate
MBC: Minimum bactericidal concentration
MDR: Multidrug resistance
MFC: Minimum fungicidal concentration
MHA: Mueller–Hinton agar
MHB: Mueller–Hinton broth
MIC: Minimum inhibitory concentration
R_*f*_: Retention factor
TLC: Thin layer chromatography
UV: Ultraviolet
ZI: Zone of inhibition.
